# The physical-mental health interface during pregnancy planning

**DOI:** 10.1192/j.eurpsy.2022.296

**Published:** 2022-09-01

**Authors:** C. Tosh, K. Kavanagh, A. Flynn, S. White, R. Catalao, C. Wilson

**Affiliations:** 1 University of Strathclyde, Mathematics And Statistics, Glasgow, United Kingdom; 2 King’s College London, Department Of Women’s Health, London, United Kingdom; 3 King’s College London, Section Of Women’s Mental Health, London, United Kingdom

**Keywords:** preconception, pregnancy, health behaviour, mental disorder

## Abstract

**Introduction:**

The physical and mental health of women prior to conception can have a significant impact on pregnancy and child outcomes. Given the rising burden of non-communicable diseases, the aim of this analysis was to explore the relationship between mental health, physical health and health behaviour in women planning a pregnancy.

**Objectives:**

To investigate the association between indices of physical and mental health in a large population of women in the UK planning a pregnancy.

**Methods:**

Responses to a preconception health digital education tool provided data on the physical and mental health and health behaviour of 131,182 women planning pregnancy. Logistic regression was used to explore associations between mental health and physical health variables. Multiple imputation by chained equations was implemented to handle missing data.

**Results:**

There was evidence for an association between physical and mental health conditions (OR 2.22; 95% CI 2.14, 2.3). There was also an association between having a mental disorder and physical inactivity (OR 1.14; 95% CI 1.11, 1.18), substance misuse (OR 2.4; 95% CI 2.25, 2.55) and less folic acid use (OR 0.89; 95% CI 0.86,0.92).

**Conclusions:**

There is a need for greater integration of physical and mental healthcare for women in the preconception period, which could support women, including those who wish to conceive, to optimise their health during this time.

**Disclosure:**

No significant relationships.

